# Supplementary material for: Serum chemerin levels: A potential biomarker of joint inflammation in women with rheumatoid arthritis
Source: PLoS One. 2021 Sep 10;16(9):e0255854. doi: 10.1371/journal.pone.0255854 (PMC8432803; doi:10.1371/journal.pone.0255854)
Supplement: Attachment — Submitted filename: Response to Reviewers ID PONE-D-21-15276docx.docx [file pone.0255854.s001.docx]

Guadalajara, Mexico July 09^th^, 2021

**Dr Emily Chenette**

**Editor-in-Chief**

**Plos One**

Ref: **Submission ID PONE-D-21-15276**

Dear Dr **Emily, Chenette:**

We are submitting the new version of the manuscript ID ab5f7e56-9b4a-4376-b0d9-1592d605fd7a entitled: “**Serum chemerin levels: a potential biomarker of joint inflammation in women with rheumatoid arthritis**”, containing all the modifications that were suggested by the Reviewers’. All the comments and corrections suggested by the Reviewers were considered point by point and the modifications were highlighted in yellow color in this new version of the manuscript.

The questions/corrections suggested by the Reviewers and the answers/modifications to those questions are described as follows:

**Reviewer 1**

1.**Reviewers’ comment:** Many statements in the INTRODUCTION section contains a scientific fact that need a reference. Please, add a reference after each statement that cite a finding.
**Authors’ response:** In accordance with the suggestion described above, we have added in the Introduction section (page 5) a reference after each statement that cite a finding. Thank you for this suggestion.

2. **Reviewers’ comment:** Exclusion criteria are not sufficient! Do author exclude patients with T2DM, hypertension, CVD,…..etc.

**Authors’ response:** In Methods section (page 7), we included in the study the patients with diabetes mellitus and hypertension. We decided to include these patients with comorbidities and then to analyze in the statistical tests if the chemerin levels were influenced by these comorbidities. In the table 1 we show a comparison of the frequency for these comorbidities. In the exclusion criteria, we have identified that RA patients with other cardiovascular diseases (history of ischemic cardiopathy, myocardial infarction and stroke) were not included.

3. **Reviewers’ comment:** Why the means and standard deviation have no decimals? Usually means and SD contain decimals. Also, p-values should contain 3 decimals.

**Authors’ response:** In accordance with the suggestion, in the Results section (page 13) we now have described in the tables and the text means and standard deviations with their decimals. We have described the p values with 3 decimals.

4. **Reviewers’ comment:** In Table 1, p-values should be re-calculated as p-value= 1 is not consistent with the results. Other p-values also are incorrect.

**Authors’ response:** We have reviewed the p-values. In the Results section (page 14), we substitute the p-value=1 by the term “NC” (not calculated). All the p-values in this new version of the manuscript contain 3 decimals.

5. **Reviewers’ comment:** In Table 2, p-values also wrong and inconsistent with the r-values. I strongly suggest re-stating the results or consulting an expert in statistics because the results are wrong!

**Authors’ response:** We have checked the accuracy of the p values, and these were consistent with the r-values. An expert in statistics was consulted, and the results were corroborated. The p values show the probability that the results were obtained by chance; whereas the r values observed in our correlations denotate the strength of the linear relationship between two quantitative variables assessed. Thank you for your comment.

6. **Reviewers’ comment:** Please, add the negative error bars in Figure 2.

**Authors’ response:** We have added the negative error bars in Figure 2. Thank you for the suggestion.

7. **Reviewers’ comment:** In Table3, Multivariate analysis usually contain Beta (SE) results in the tables. It is not necessary to add “enter method” or stepwise method. Add the decimals to the p-values.

**Authors’ response:** In the Results section, we have added in the Table 3 (page 17), the values of Beta and Standard Error. We have included in the multivariable analysis presence of diabetes mellitus as comorbid. We have removed of the columns the terms “enter method” or “stepwise method”. We have added the 3 decimals to the p-values. Thank you for these recommendations.

8. **Reviewers’ comment:** Do author make a screening for selection a patient from a specific city or hospital? If yes, Figure 1 is necessary. If not, Figure 1 is not necessary.

**Authors’ response:** We have included in the Figure 1, the information of the specific hospital where the RA patients were selected. Thank you for your suggestion.

**Reviewer 2**

**1.Reviewers’ comment:** The abstract should be re-checked and ameliorated, especially the sentences about the conclusions…. which are not the conclusion of the manuscript.

**Authors’ response:** We have rewritten the abstract (page 3 and 4). We have modified the conclusion. Now there is a concordance between the conclusion in the abstract and the conclusion in the text of the manuscript (page 22).

2. **Reviewers’ comment:** The introduction is complete and clear, but the objective of the study should be more impressive…

**Authors’ response:** In the Introduction section (page 6), we have modified the objective of the study according to the comments. Now the new objective is described as follows: “Therefore, the objective of this study was to evaluate whether serum chemerin is an independent biomarker of moderate or severe disease activity in RA patients”. We thank for this important suggestion.

3. **Reviewers’ comment:** Concerning method section, the authors should include the description of all the considered demographic and clinical characteristics of the patients enrolled in the study… as well as the inclusion and exclusion criteria, comorbidities, other pharmacological treatments….

**Authors’ response:** In the Method section (page 7), we have modified the inclusion criteria, to include a more detailed description of the inclusion and exclusion criteria, comorbidities, and pharmacological treatments.

4. **Reviewers’ comment:** In the results, please describe the flowchart in a better way.

**Authors’ response:** In the Results section (page 10), we have described in more detail the flowchart.

5. **Reviewers’ comment:** In table 1 please add all the data analyzed.

**Authors’ response:** In the Results section (page 13 and 14), we have added in table 1, all the data analyzed. Thank you for your suggestion.

6. **Reviewers’ comment:** Please develop the conclusions of the study. The Authors should consider what all of the findings taken together mean, what are the larger implications.
They also need to clarify what advance or advances in knowledge were made by this work.

**Authors’ response:** We have rewritten the conclusion of the study, in the text (page 22) and in the Abstract section (page 4).

7. **Reviewers’ comment:** What all of the findings taken together mean, what are the larger implications.
They also need to clarify what advance or advances in knowledge were made by this work.
**Authors’ response: We also added relevant information regarding the interpretation of our findings in the paragraph related with the importance of our study i**n the Discussion section (page 21), “The present study identified a relationship between high chemerin levels and the severity of disease activity in RA patients; therefore, the results of this work generate new questions about the role of chemerin in the persistence of the inflammatory process in RA. Chemerin is an adipokine synthesized mainly in adipose tissue and liver, and diverse immune cell subsets, including plasmacytoid dendritic cells, macrophages and NK cells, express the chemerin receptor CMKLR1, which when activated promotes chemotaxis and modulates the immune response. In the present study, we identified an association between chemerin levels and CRP and the number of swollen joints; these findings indicate the relevance of chemerin in inflammation in RA”. Thank you for the suggestion.

**8.** **Reviewers’ comment:** Check the main text, there are some mistake in English language.

**Authors’ response:** The manuscript has been checked and corrected. The English language editing certificate was issued on June 28, 2021 and may be verified on the AJE website using the verification code 216B-E352-B7D8-E106-F506. Thank you for the suggestion.

**Dr Laura Gonzalez-Lopez**

Author for correspondence

**Primary email:** [lauraacademicoudg@gmail.com](mailto:lauraacademicoudg@gmail.com)

**Alternative email:** [dralauragonzalez@prodigy.net.mx](mailto:dralauragonzalez@prodigy.net.mx)

**Ernesto German Cardona**

Author for correspondence

**Email:** [cameg1@gmail.com](mailto:cameg1@gmail.com)
